# Public health and economic impact of vaccination with 7-valent pneumococcal vaccine (PCV7) in the context of the annual influenza epidemic and a severe influenza pandemic

**DOI:** 10.1186/1471-2334-10-14

**Published:** 2010-01-21

**Authors:** Jaime L Rubin, Lisa J McGarry, Keith P Klugman, David R Strutton, Kristen E Gilmore, Milton C Weinstein

**Affiliations:** 1 i3 Innovus, 10 Cabot Road, Suite 304, Medford, MA 02155, USA; 2 Department of Global Health, Rollins School of Public Health, Emory University, 1518 Clifton Road, N.E - Room 720, Atlanta, GA 30322, USA; 3 Pfizer, 500 Arcola Road, Collegeville, PA, 19426, USA; 4 Harvard School of Public Health, Harvard University, 718 Huntington Avenue, Boston, MA 02115, USA

## Abstract

**Background:**

Influenza pandemic outbreaks occurred in the US in 1918, 1957, and 1968. Historical evidence suggests that the majority of influenza-related deaths during the 1918 US pandemic were attributable to bacterial pneumococcal infections. The 2009 novel influenza A (H1N1) outbreak highlights the importance of interventions that may mitigate the impact of a pandemic.

**Methods:**

A decision-analytic model was constructed to evaluate the impact of 7-valent pneumococcal conjugate vaccine (PCV7) on pneumococcal disease incidence and mortality during a typical influenza season (13/100) and a severe influenza pandemic (30/100). Outcomes were compared for current PCV7 vaccination practices vs. no vaccination. The model was estimated using published sources and includes indirect (herd) protection of non-vaccinated persons.

**Results:**

The model predicts that PCV7 vaccination in the US is cost saving for a normal influenza season, reducing pneumococcal-related costs by $1.6 billion. In a severe influenza pandemic, vaccination would save $7.3 billion in costs and prevent 512,000 cases of IPD, 719,000 cases of pneumonia, 62,000 IPD deaths, and 47,000 pneumonia deaths; 84% of deaths are prevented due to indirect (herd) protection in the unvaccinated.

**Conclusions:**

PCV7 vaccination is highly effective and cost saving in both normal and severe pandemic influenza seasons. Current infant vaccination practices may prevent >1 million pneumococcal-related deaths in a severe influenza pandemic, primarily due to herd protection.

## Background

Pandemic influenza outbreaks occurred in the US in 1918, 1957, and 1968, and the World Health Organization declared the novel influenza A (H1N1) virus a pandemic in June 2009. The most notable pandemic occurred in 1918 (H1N1 strain) and caused at least 20 million deaths worldwide, with some estimates as high as 100 million [1-4]. Infection rates (25 - 50% of the population) and deaths (up to 2%) were much higher than is typically observed in an influenza outbreak [5]. A severe influenza pandemic similar to 1918 would have devastating effects in the US and globally, with an estimated 90 million cases of influenza, 1.9 million deaths, 9.9 million hospitalizations, 42 million outpatient visits, and costs of up to $255 billion in the US alone [6].

The unprecedented severity of the 1918 influenza pandemic has led to much research, particularly on the high burden of mortality in otherwise healthy young adults. Experts agree that influenza alone cannot explain the extraordinary number of pandemic deaths, but attach different emphasis to the role of secondary bacterial pneumonias. More recent studies support the conclusions of investigators at the time of the pandemic [7] that most deaths were due to complications of bacterial pneumonia. A study of autopsy samples from victims of the 1918 pandemic found bacteria in all samples, pointing to bacterial pneumonia as the cause of death [8]. Further, an analysis of the time to death from pneumonia associated with influenza in 1918 shows concordance with contemporary accounts of time to death from pneumococcal pneumonia [9]. A recent autopsy study of H1N1 victims found bacterial pneumonia in 55% of their samples [10]. These observations are further supported by a recent double-blind randomized trial of pneumococcal conjugate vaccine in children not vaccinated for influenza that demonstrated a 45% lower incidence of hospitalization due to influenza-associated pneumonia among those receiving the pneumococcal vaccine [11].

In 2000, the 7-valent pneumococcal vaccine (PCV7; Prevenar; Wyeth Pharmaceuticals, acquired by Pfizer in October 2009) was approved for pediatric use and recommended by the Centers for Disease Control and Prevention (CDC) for children aged up to 59 months [12]. Routine infant vaccination with PCV7 (administered at 2, 4, 6, and 12 to 15 months of age) [13] has markedly decreased the incidence of invasive pneumococcal disease (IPD) in both the vaccinated and general populations [14-16]. CDC analyses from 2005 indicate that most IPD (69%) was prevented through indirect (herd) effects of the vaccine [17], likely due to decreased nasopharyngeal carriage of pneumococcal strains among immunized children, resulting in decreased transmission to non-immunized children and adults [17]. The impact of PCV7 on pneumococcal disease - including pneumonia [18] and IPD [19] - in the unvaccinated population, and the likely synergies between pneumococcal disease and influenza, suggest current PCV7 vaccination practices may reduce morbidity and mortality in an influenza pandemic. While influenza vaccination remains the most effective means of reducing influenza-related morbidity and mortality, the risk of influenza and pneumococcal disease co-infection in a pandemic, along with the ability to vaccinate individuals prior to the onset of a pandemic, provide strong rationale for considering pneumococcal vaccination in the context of influenza. This study examines the public health and economic impact of current pneumococcal vaccination policies in the context of the annual influenza epidemic and a severe influenza pandemic.

## Methods

### Overview

We used a decision-analytic model of pneumococcal disease incidence and outcomes to assess the impact of current US pneumococcal vaccination practices on pneumococcal disease burden during both an annual influenza epidemic and a severe influenza pandemic, relative to the hypothetical burden if no pneumococcal immunization program had been implemented. The model considers outcomes of pneumococcal disease only; morbidity, mortality and costs of influenza without pneumococcal co-infection are not considered. Outcomes include cases of pneumococcal disease, pneumococcal-related deaths, costs, survival (in life-years [LYs]) and quality-adjusted survival (in quality-adjusted life-years [QALYs]). To simulate a single influenza season, epidemiologic outcomes were calculated over a 1-year period; incremental costs, survival, and quality-adjusted survival reflect lifetime consequences of events occurring during this 1-year period. Future costs and health outcomes were discounted to present values using a 3% annual discount rate [20]. This analysis presents results from a payer perspective and all costs are in 2006 dollars.

### Model Structure

**Figure 1:**
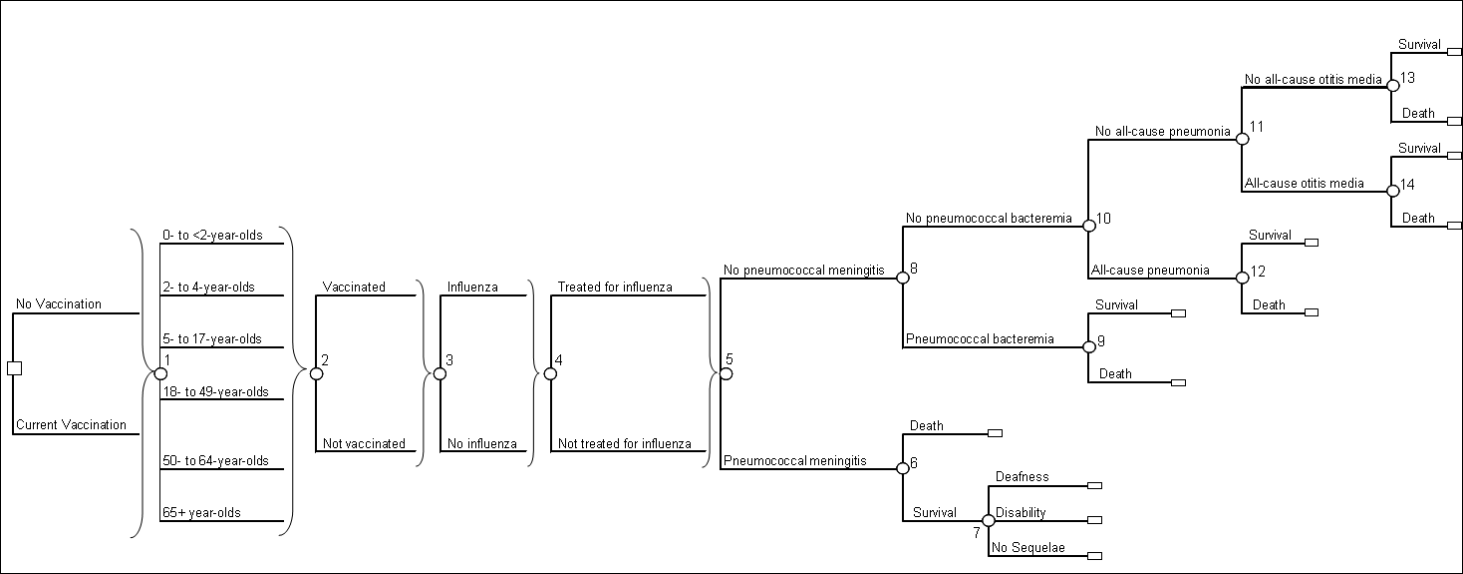
**Decision model structure**. In each age group, persons could be vaccinated or unvaccinated, depending on vaccination policy and coverage (node 2). Both vaccinated and unvaccinated persons are at risk of influenza (node 3) and may or may not receive treatment for this condition (node 4). Pneumococcal disease sub tree. In the model, persons are first subject to the risk of meningitis (node 5), which can lead to death (node 6) or to deafness, disability, or no sequelae (node 7). Those not experiencing meningitis are subject to the risk of bacteremia (node 8), including bacteremic pneumonia, which may lead to death (node 9). Similarly, persons who do not contract meningitis or bacteremia are then at risk for non-bacteremic pneumonia (node 10) and AOM (node 11), and they can die of pneumonia (nodes 12). Persons may also die of causes unrelated to pneumococcal disease; these deaths are incorporated into the event-specific mortality probabilities (at nodes 6, 9, 12, and 14), and are captured separately for those who avoid acute pneumococcal events (node 13).

Figure 1 shows clinical events relating to vaccination and influenza incidence and treatment; the model population is stratified into six age groups: 0 to <2 years, 2 to 4 years, 5 to 17 years, 18 to 49 years, 50 to 64 years, and >65 years. Subsequent branching of the tree highlights potential health outcomes of pneumococcal disease and the possibility of developing meningitis, bacteremia, pneumonia, or acute otitis media (AOM). Consistent with other economic models [21,22] and the Northern California Kaiser Permanente (NCKP) trial [23], meningitis and bacteremia are defined as those caused by *S. pneumoniae*, whereas pneumonia and AOM cases are caused by any organism (all-cause). For simplicity, the clinical manifestations of pneumococcal disease are modeled as mutually exclusive, with risk of occurrence following a hierarchy of severity from most severe to least severe: meningitis, bacteremia, pneumonia, AOM.

### Model Estimation

Input parameters used in the model and corresponding data sources are presented in Table 1.

**Table 1:** Input parameters

	Age group (years)					
	0 - <2	2 - 4	5 - 17	18 - 49	50 - 64	65+
**Annual Incidence per 100,000**						
Pneumococcal meningitis^/a^	9.1	1.1	2.3	0.5	1.5	1.7
Pneumococcal bacteremia^/a^	174.8	35.8	3.8	12.3	22.7	58.8
All-cause pneumonia^/b^	4,710	1,517	329	383	1,462	9,294
All-cause otitis media (per person)^/c^	1.10	0.58				
**Case Fatality Rates**						
Pneumococcal meningitis^/d^	0.05	0.05	0.04	0.13	0.21	0.27
Pneumococcal bacteremia^/d^	0.01	0.01	0.02	0.08	0.12	0.16
All-cause pneumonia^/e^	0.00	0.02	0.02	0.02	0.05	0.05
**Proportion meningitis cases resulting in (%)**						
Deafness^/f^	13.0	13.0	6.4	13.0	13.0	13.0
Disability^/f^	6.7	6.7	5.2	6.7	6.7	6.7
**Vaccine Effectiveness**						
IPD						
Efficacy^/g^	73.5%	67.0%				
Indirect (herd) effect^/h^	46.8%	40.3%	17.5%	38.3%	17.4%	33.6%
All-cause pneumonia						
Efficacy^/i^	6.9%	6.3%				
Indirect (herd) effect^/j^	17.6%	18.9%	9.0%	13.0%	9.3%	7.7%
All-cause otitis media						
Efficacy^/i^	6.4%	5.8%				
Indirect (herd) effect^/k^	20.1%	19.0%				
**Costs ($) per Case of Pneumococcal Disease**						
Meningitis^/l^	13,196	13,196	7,446	10,586	13,461	10,263
Deafness^/m^	101,975	101,387	97,679	82,278	57,428	31,733
Disability^/m^	526,174	523,143	504,006	424,543	296,317	163,738
Bacteremia^/l^	2,754	2,754	7,446	10,586	13,461	10,263
Pneumonia^/n^	592	592	5,166	6,465	7,558	7,263
Otitis media^/o^	256	256				

Indirect (herd) effect refers to percent reduction in disease incidence in the unvaccinated

IPD = invasive pneumococcal disease

a. Incidence of pneumococcal meningitis and bacteremia were estimated from the published ABCs report [15]. The ABCs reports incidence for 18-34 and 35-49 year old persons separately; we combined these age groups using weighted averages based on census data.

b. Incidence rates for all-cause pneumonia were adapted from Ray et al., which used unpublished Kaiser Permanente data to estimate incidence in unvaccinated populations [21,26].

c. Incidence of AOM adapted from Ray et al., combining simple and complex AOM. Only children <5 years were assumed to be at risk for AOM [21].

d. Case-fatality rates for IPD were estimated from the published ABCs report [14,15] and Robinson et al. [33]; we divided the incidence reported in the ABCs by the age-specific mortality reported by Robinson et al.

e. Case-fatality rates for pneumonia were estimated from a study of community-acquired pneumonia [31]. It was assumed that there was no risk of death from AOM.

f. The probabilities of deafness and disability due to meningitis were adapted from data in children and adolescents with bacterial meningitis [22,27-29].

g. Adapted from the NCKP trial of PCV7 [23]; 94% (intent-to-treat) efficacy against covered serotypes, with PCV7 coverage of approximately 80% against *S.pneumoniae *serotypes that cause pneumococcal meningitis and bacteremia.

h. In children <5 estimated as the difference between observed changes in disease incidence from the (ABCs) Report and direct vaccine efficacy from the NCKP trial, assuming all changes in incidence not attributable to vaccine efficacy were attributable to indirect (herd) effects [14,15,23]; in adults estimated based changes in incidence reported in the ABCs report.

i. Based on intent-to-treat data from the NCKP trial for PCV7 [23].

j. In children < 5, estimated from assuming overall effectiveness is the midpoint between the NCKP trial data, which reported vaccine efficacy of 6.9% in all-cause pneumonia [26] and ecologic data reported by Grijalva (39% reduction) [18] and assuming all reductions in disease not attributable to vaccine efficacy are attributable to indirect (herd) effects. We chose the midpoint because it is not known what proportion of the reduction in admissions reported by Grijalva were due to the direct effects of PCV7 versus indirect (herd) effects within the vaccine-eligible population, and these ecological data reflect changes in hospital admissions for pneumonia rather than incidence; final estimates of indirect (herd) effects against pneumonia were similar in magnitude to the reduction in x-ray confirmed pneumonia from the trial [33,34]. In adults, indirect (herd) effects against pneumonia were estimated from ecologic data reported by Grijalva et al. [18].

k. Efficacy against AOM was calculated in a manner similar to that of pneumonia; we used the midpoint between the NCKP trial estimate (6.4%) [23] and results from a study that examined changes in AOM-related outpatient visits before and after the introduction of PCV7 (42.7% reduction) [32]; our assumption of the estimated proportion of the reduction in AOM cases that is biologically plausible to be attributable to PCV7 was based on expert opinion (personal communication with Keith Klugman, MD, PHD, Steve Pelton, MD, and Michael E. Pichichero, MD).

l. The cost of diagnosing and treating meningitis and bacteremia, were derived from Ray et al. [21,22]. Because this study did not report costs of meningitis and bacteremia separately for persons >5 years of age, we assigned the reported cost to both meningitis and bacteremia in these age groups.

m. Costs of long-term consequences of meningitis were adapted from lifetime costs of deafness and disability for children <5 years [21]. To calculate the lifetime costs in persons aged >5 years, we multiplied the costs for children <5 years by the proportional difference in discounted life-expectancy as estimated from US life-tables [45].

n. We combined costs of hospitalized pneumonia for persons >5 years of age [21] and non-hospitalized community-acquired pneumonia [45] to calculate the overall cost of all-cause pneumonia, assuming hospitalization rates of 12% for those aged 5 to 17 years, 28% for 18- to 49-year-olds, 25% for those aged >50 [18,21,47].

o. We assumed that 7% of AOM cases are complex and 1.4% of cases required tympanostomy tube placement [22], and estimated the cost of simple AOM as $192, complex AOM as $557, and tympanostomy tube placement as $2,687 [21]. The reported cost is a weighted average of simple and complex AOM and cases requiring tube placement.

#### Population, Vaccine Coverage, Incidence & Mortality

The age group distribution of the US population was estimated from the 2006 US Census [24]. Current vaccination practices are defined based on 2006 data from the National Immunization Survey (NIS) as 87% of children <2 years vaccinated with PCV7; 79% of these children receive 4 doses, and the remainder 3 doses. We assumed that children aged 2 to 4 years were vaccinated an average of 2 years prior to the start of the model, therefore, NIS data from 2004 were used to estimate the proportion of children in this age group who retained vaccine protection (69% of those receiving 3 or more doses and 71% of those receiving 4 doses) [25]. Age-specific disease incidence rates before PCV7, case-fatality rates, and complication probabilities for pneumococcal disease manifestations were estimated from published sources [14,15,21,22,26-31].

#### Vaccine Efficacy & Indirect (Herd) Effects

Vaccine effectiveness (efficacy and indirect [herd] effects) estimates in all age groups are shown in Table 1. Vaccine efficacy estimates against IPD in children <5 years were based on results of the NCKP trial. In vaccinated children <2 years, vaccination was assumed to directly reduce the probability of pneumococcal infection by the recorded efficacy of the vaccine [23] for children 2 - 4 years; efficacy was reduced to reflect waning effectiveness of the vaccine over time [22]. Indirect (herd) effects were incorporated in the model as a percent reduction in disease incidence. To calculate herd effects on IPD in the vaccine-eligible population, we used differences in the observed changes in disease incidence from the Center for Disease Control and Prevention's Active Bacterial Core Surveillance (ABCs) Report and direct vaccine efficacy from the NCKP trial, assuming all changes in incidence not attributable to vaccine efficacy were attributable to indirect (herd) effects [14,15,23].

Ecologic data on pneumonia-related hospital admissions and AOM visits suggests that PCV7 vaccination may have led to larger reductions in all-cause pneumonia (39.0%) and AOM (42.7%) in children than trial efficacy data indicate [18,23,32]. We therefore estimated the effectiveness (both direct and indirect) of vaccination in children as the midpoint between the NCKP trial estimates and the ecologic data [32-34]. We then calculated the indirect (herd) effect of vaccination assuming all changes in incidence not attributable to direct protection were attributable to indirect (herd) protection and that indirect (herd) effects apply to both the vaccinated and unvaccinated populations.

Indirect (herd) effects in the unvaccinated age groups were calculated from observed reductions in IPD incidence [14,15] before and after the introduction of PCV7 [21]; all reductions in disease were assumed to occur in the 7 serotypes included in the vaccine. Because it is not known what proportion of the observed reduction in pneumonia is attributable to the vaccine, and regional analyses have not supported changes observed in national datasets [26,35], we assumed that effects in the unvaccinated were equal to 50% of the estimated effect reported by Grijalva et al. [18].

#### Influenza

One-year cumulative influenza incidence was calculated from the CDC's Flu Activity & Surveillance Weekly Activity Reports (13%) [36,37]. It was assumed that persons with influenza were at a higher risk of pneumococcal disease than those without influenza. Persons without influenza were considered the reference group and those with influenza were assumed to have 7.7% increased risk of pneumococcal disease; the increased risk was calculated from a study that examined excess pneumococcal infections during a normal influenza season (RR: 1.077) [38]. Base-case probabilities of pneumococcal disease were adjusted to reflect the non-pandemic relative risk for persons with versus without influenza; therefore overall rates of disease in a non-pandemic year are equal to incidence rates from the literature. We assumed that persons treated for influenza were at 26.3% lower risk of pneumococcal disease relative to untreated persons based on the reduction in duration of influenza from a trial of oseltamivir versus placebo [39,40]. The proportion treated for influenza was estimated from published sources examining treatment patterns for influenza [41-44].

#### Costs & Utilities

All costs were estimated from published sources and converted to 2006 US dollars using the Consumer Price Index [18,21,22,45-49]. The average price for a single dose of PCV7 was assumed to be $66.50 [48] with an administration cost of $9.92 [21]. To account for the 5-year benefit of vaccination, we amortized the cost over a 5-year period, discounting all future years by 3%. Utility values, measures of quality-of-life ranging from 0 to 1 used to calculate QALYs, were adapted from a cost-effectiveness analysis of pneumococcal vaccination [50]. Utilities associated with acute disease states were incorporated into the model by assigning a "toll" in the form of an absolute QALY decrement to each episode of illness. We applied a decrement of 0.023 for meningitis, 0.008 for bacteremic pneumonia, 0.006 for hospitalized pneumonia, 0.004 for non-hospitalized pneumonia, and 0.005 for AOM to all age groups. Utilities for chronic states (deafness and disability) were estimated from retrospective studies of meningococcal complications as 0.73 for deafness and 0.68 for disability [51,52] and applied to the remaining life expectancy.

#### Pandemic Calibration

To test the assumption that synergies between influenza and pneumococcal disease observed during a normal influenza season are magnified during a pandemic [53-55], we increased the incidence of influenza in our model to 30%, which is consistent with expected incidence of influenza in a pandemic in the US [2]. However, we were unable to replicate the increased incidence of pneumococcal disease seen during the 1918 pandemic. To estimate the effect of influenza on pneumococcal disease incidence during a severe pandemic, we calibrated the increased relative risk of pneumonia (bacteremic and non-bacteremic) for those with influenza versus those without to achieve the 5% incidence of combined infection with influenza and pneumonia observed in 1918 [56]. To estimate age-specific influenza and pneumonia incidence, we applied estimates of age-specific excess population mortality in 13 countries, and scaled the pneumonia and influenza incidence in proportion to the observed excess pneumococcal and influenza-related mortality in the US (0.39%), assuming 30% of persons had influenza [57]. When performing the calibration, we also assumed that 30% of pneumonia cases were bacteremic in a severe pandemic [55]. The largest increase in incidence was observed among influenza cases in the 18 - 49 year age group (4-fold). Due to the introduction of antibiotics, it was deemed unlikely that the next pandemic would result in the excess mortality of 0.39% seen in the US during the 1918 pandemic. We assumed that case-fatality rates for IPD and pneumonia during a severe pandemic would instead be similar to those of persons on antibiotic therapy (10% for all-cause pneumonia in all age groups and 10% for IPD for those <50 years) [58]. The case-fatality for those >50 years was assumed to be equal to the case-fatality rates in a normal influenza season.

### Analyses

#### Base-case Analyses

Base-case analyses assumed a population of 300 million people. It was assumed that 7.2 million infants (<2 years of age) were fully vaccinated at the start of the model, and that 8.5 million children 2 - 4 years of age had been vaccinated prior to the start of the model and were still receiving immunological benefits. Unvaccinated persons benefited from vaccination via indirect (herd) effects. The model was used to calculate cases of pneumococcal disease avoided, deaths averted, QALYs gained, LYs saved, and costs. Cases avoided and deaths averted were calculated as the difference in the total number of cases/deaths under a policy of no vaccination minus the total number of cases/deaths under current vaccination practices. Total costs with vaccination were calculated as the cost of vaccination less any cost offsets associated with avoiding disease.

Incremental cost-effectiveness ratios (ICERs) were estimated in terms of additional cost per QALY gained or additional cost per LY saved, and calculated by dividing the difference in costs by the difference in QALYs or LYs. LYs saved for each death averted were calculated using the discounted life expectancy at each age. QALYs gained were calculated based on LYs saved, first converting discounted life expectancy at each age into quality-adjusted life expectancy, then weighting each discounted year of life by an age-specific utility weight for healthy individuals [59]. Lifetime consequences of meningitis were included by assigning the utility associated with meningitis sequelae for the duration of age-specific discounted life-expectancy and subtracting the resulting quality-adjusted life-expectancy from the quality-adjusted life-expectancy without meningitis sequelae. Finally, to account for acute illness disutility, QALY decrements were subtracted from quality-adjusted life-expectancy. All costs and life years were discounted at 3% per annum. Analyses were conducted under conditions of a normal influenza season (influenza incidence of 13%) and a severe pandemic season (influenza incidence of 30%).

#### Sensitivity Analyses

Because the pneumonia and influenza incidence distribution of a future pandemic is uncertain, we re-calibrated the pandemic parameters to reflect the current age distribution of pneumococcal disease. The overall calibration target remained at 5% incidence of combined pneumonia and influenza; however, we re-distributed the burden such that persons >65 years and <2 years had the highest incidence of pneumococcal disease and those aged 5 - 49 years the lowest. Case-fatality rates remained unchanged from the base-case pandemic analysis.

A series of one-way sensitivity analyses were performed to assess the robustness of model results to plausible alternative assumptions regarding input parameters. The proportion of the observed reduction in pneumonia post-introduction of PCV7 that is attributable to PCV7 is uncertain; we varied the indirect (herd) effect on pneumonia from zero to the values reported by Grijalva et al. [18]. Because treatment patterns for influenza may change during a pandemic, we varied the percent of persons treated for influenza by ± 25%. To reflect the uncertainty in the incidence and mortality burden of a future pandemic, we re-estimated the model assuming that bacteremic pneumonia incidence and case-fatality were reduced by 50% from the base-case. To account for the effect of possible antibiotic shortages during a pandemic, case-fatality rates for bacteremic and all-cause pneumonia were increased to 20 percent. Other sensitivity analyses included: [1] reducing assumed effectiveness of pneumococcal vaccination on AOM to 6% (reported in the NCKP trial [23]) and increasing effectiveness to the 42.7% reported by Zhou et al. [32]; [2] varying price of the vaccine by ± 10%; [3] varying incidence of invasive disease by ± 10%; and [4] varying vaccine coverage rates by ± 10 percent. Sensitivity analyses are reported for pandemic conditions only.

## Results

### Non-pandemic influenza season

The model predicts that current vaccination practices prevent approximately 32,300 IPD cases, 550,100 pneumonia cases, 2,200 IPD deaths, and 21,000 pneumonia deaths during a normal influenza season. Cost savings are estimated to be $1.57 billion; vaccination with PCV7 is predicted to be less costly as well as more effective than no PCV7 vaccination (i.e., dominant).

### Severe pandemic season

#### Base-case

To reach the expected excess incidence of pneumococcal disease during a severe pandemic, the relative risk of bacteremic pneumonia among persons with influenza versus without influenza was increased by a multiple ranging from 15-fold in children <2 years to 150-fold in persons aged 5 - 17 years over the estimate for a non-pandemic year. It was assumed that persons without influenza were not at increased risk of pneumococcal disease during a severe pandemic. Table 2 shows the calibrated incidence of pneumococcal disease for persons with influenza during a severe pandemic season; non-pandemic incidence rates are included as a point of reference.

**Table 2:** Incidence in pandemic and non-pandemic years

	Age group (years)					
Annual Incidence of PD per 100,000	0 - <2	2 - 4	5 - 17	18 - 49	50 - 64	65+
**Incidence of PD in a non-pandemic year (13% influenza incidence)**						
Among persons without influenza						
Bacteremic pneumonia	173.0	35.4	3.7	12.1	22.5	58.2
Other pneumonia	4,662	1,501	326	379	1,447	9,200
Among persons with influenza						
Bacteremic pneumonia	186.3	38.1	4.0	13.1	24.2	62.6
Other pneumonia	5,020	1,616	351	408	1,558	9,906
**Incidence of PD in a severe pandemic year (30% influenza incidence)**						
Among persons with influenza^/a^						
Bacteremic pneumonia	2,898	1,688	600	1,192	1,056	4,166
Other pneumonia	6,954	4,006	1,409	2,814	2,491	10,143

PD = pneumococcal disease

a. The incidence of PD among persons without influenza during a pandemic is assumed to be the same as in a non-pandemic year

Projected outcomes during a severe pandemic are displayed in Table 3. The majority of cost savings are attributable to avoiding disease in the non-vaccine-eligible age groups. Preventing pneumococcal disease in persons aged 18 - 49 or >65 years each accounts for 40% of the cost savings and accounts for 80% combined. The model predicts that pneumococcal vaccination would lead to 2.0 million LYs saved by avoiding 109,000 deaths due to IPD or pneumonia, and 2.1 million QALYs gained, in a population of 300 million. The majority of LYs saved and QALYs gained are in the 18 - 49 year age group, where vaccination is expected to lead to 841,000 LYs saved and 784,000 QALYs gained in a severe pandemic.

**Table 3:** Base-case and sensitivity analysis results

Analysis	Values	Cases avoided	Deaths averted	Cost (savings) in billions of $
**Base-case**	**--**	**4,726,000**	**108,500**	**(7.34)**

Re-calibrated using current non-pandemic incidence distribution by age	--	4,430,000	73,100	(4.8)

Incidence and case-fatality of bacteremic pneumonia - reduced by 50%	--	3,873,000	39,600	(4.09)

Case-fatality rates for IPD and pneumonia - increased to 20%		--	201,000	(7.33)

Herd effect on pneumonia				
Low (0%)	0%	4,106,000	59,600	(5.61)
High^16^	15% - 26%	5,345,000	157,300	(9.07)

Incidence of IPD (per 100,000)				
Low (-10%)	296 - 8,365	4,689,000	102,700	(6.77)
High (+10%)	383 - 10,224	4,763,000	114,200	(7.90)

Incidence of all-cause pneumonia (per 100,000)				
Low (-10%)	5 - 165	4,681,000	103,700	(7.17)
High (+10%)	7 - 202	4,770,000	113,300	(7.50)

Vaccine effectiveness on AOM (<2 Yr)				
Low^21^	6%	2,687,000	--	(6.81)
High^27^	42%	10,444,000	--	(8.80)

Vaccine coverage (<2)				
Low (-10%)	78%	4,661,000	107,900	(7.40)
High (+10%)	96%	4,790,000	109,000	(7.27)

Price of vaccine				
Low (-10%)	$68.78	--	--	(7.44)
High (+10%)	$84.06	--	--	(7.24)

Influenza treatment				
Low (-10%)	8% - 16%	4,728,000	--	(7.34)
High (+10%)	14% - 26%	4,725,000	--	(7.34)

Case-fatality from IPD				
Low (-10%)	0.8% - 25%	--	102,400	--
High (+10%)	0.9% - 30%	--	114,500	--

Case-fatality from all-cause pneumonia				
Low (-10%)	0.4% - 4.7%	--	103,700	--
High (+10%)	0.5% - 6%	--	113,300	--

IPD = invasive pneumococcal disease; AOM = acute otitis media

#### Sensitivity Analyses

In addition to the base-case estimates, table 3 shows the sensitivity of pneumococcal disease cases, deaths, and costs to variations in model inputs in a severe pandemic influenza season. Redistributing the disease burden to reflect current age distributions of disease incidence results in fewer cases of IPD avoided due to less indirect (herd) effect against bacteremic pneumonia in the elderly when compared with younger adults (18 - 49 years).

Model results are sensitive to reductions in the incidence and case-fatality rates of bacteremic pneumonia and all-cause pneumonia, which lead to substantial cases and deaths averted as well as cost savings. Assuming additional herd protection against pneumonia at the levels reported by Grijalva et al. [18] or additional vaccine effectiveness against AOM [32], substantially increases cost savings. Assumed changes in vaccination practices as well as small changes (± 10%) in the percentage of persons receiving treatment for influenza, the case-fatality from IPD or pneumonia, and the price of the vaccine have minimal effects on model results.

## Discussion

To our knowledge, no other studies have evaluated the economic impact of vaccination with PCV7 during an influenza pandemic. Studies have examined the cost-effectiveness of PCV7 against pneumococcal disease [21,22,50,60-63]. However, these studies did not examine PCV7 in the context of influenza. Results of our study are consistent with studies examining other interventions to reduce the epidemiologic and economic impact of an influenza pandemic. For example, studies in Israel [64], Singapore [65] and the UK [66] found stock-piling antiviral drugs to be either cost-effective or cost saving in a pandemic. One study assessed vaccination with a 23-valent pneumococcal vaccine in the context of an influenza pandemic in the Netherlands. The study found that in the absence of an available influenza vaccine at the start of a pandemic, pneumococcal vaccination should be administered to the elderly and groups at a high risk for influenza, in order to limit the number of resulting hospitalizations and deaths [67].

Several conservative assumptions regarding the effects of pneumococcal vaccination and disease epidemiology were made. We assumed no direct protection against pneumococcal disease after 5 years of age. While it is unlikely that the vaccine will wane to zero when children turn age 5, data are not available to characterize the degree of waning vaccine efficacy in this age group over time. We therefore made assumptions consistent with previously published cost-effectiveness models of PCV7 [21]. The pneumonia (bacteremic and non-bacteremic) case-fatality rate of 10% during a pandemic assumes access to antibiotics would not be disrupted; however, it is likely that access may be restricted due to increased demand and subsequent shortages. We also included conservative estimates of indirect (herd) effects against AOM and pneumonia in the vaccine-eligible population, taking the midpoint of observed vaccine efficacy in the NCKP trial and the observed reductions in hospital admissions from a community-based study [18,26,32]. Even under these conservative assumptions, the base-case outputs predict a large positive public health and economic impact of vaccination.

Our study is subject to a number of limitations inherent in the study design. First, the decision-analytic model is necessarily a highly simplified representation of the disease transmission and outcomes of pneumococcal disease. Although we accounted for some differences in treatment and outcomes using age stratification, we recognize that the US population and health-care delivery system is highly heterogeneous and may not be well represented by the relatively simple structure of this model. We also note that data used to estimate vaccine effectiveness and outcomes were derived and synthesized from a variety of sources, and this process of interpretation and decision-making is subject to bias. Although extensive sensitivity analyses to evaluate the effect of alternative parameter choices on our outcomes showed no change in the overall conclusions, we recognize that different assumptions may have yielded different results. Costs used in this analysis were taken from published data and standard sources; the extent to which they reflect the true costs of administering medical care is unknown. Furthermore, this study was conducted from a third-party payer perspective rather than a societal perspective, and as such does not include costs of pneumococcal disease related to lost productivity, caregiver time, transportation, or other unreimbursed expenses. Inclusion of indirect costs presumably would have added substantially to the total cost burden of pneumococcal disease and the potential cost savings with PCV7. In addition, the model was estimated using US data, and care should be used in generalizing our results to other settings and populations.

Because the disease and mortality burden have differed both in magnitude and age distribution during the four pandemics that have occurred in the past century, it is difficult to characterize a "typical" pandemic. Our assumptions derived from the 1918 pandemic likely reflect a relatively severe influenza pandemic scenario. Preliminary novel influenza A (H1N1) pandemic data indicate that it is less virulent than previous pandemic strains, with fewer required hospitalizations, and deaths more likely to be concentrated in those with underlying medical conditions [68]. There is also evidence that the presence of secondary bacterial infections in persons with influenza is lower in the 2009 pandemic relative to the 1918 pandemic [10,69], indicating that the novel H1N1 disease burden will likely fall between that of a normal season and a severe pandemic. Because PCV7 vaccination is beneficial under both the severe pandemic and seasonal epidemic scenario, we can infer that PCV7 would also result in cost savings and public health benefits in a less severe pandemic. Future analyses assessing the impact of pneumococcal vaccination in the context of the novel influenza A (H1N1) pandemic will be undertaken as more data become available.

We acknowledge that access to care and antibiotics, as well as volume and means of travel have changed dramatically since 1918. However, under various assumptions meant to capture how access to care, antibiotic use, and differences in disease transmission may alter disease dynamics (i.e., reducing incidence and mortality during a pandemic), pneumococcal vaccination still remains highly cost saving. We also acknowledge that this 1-year model estimated from 2006 data includes the indirect (herd) effects of a national immunization program with PCV7 that has been established over 6 years, and only accounts for the costs of vaccination of 4 birth cohorts (children aged 1 year, 2 years, 3 years, and 4 years). In order to quantify the current value of the ongoing vaccination program, we chose to include the full effects of vaccination. If we had accounted for the costs and benefits over each year since the launch of PCV7, the economic argument likely would be diminished, as the indirect benefit grew over the 4 - 6 years after the launch of the vaccine. However, even if we assume that only half of the indirect benefit had accrued after 4 years of vaccination, the model predicts that vaccination would avoid >700,000 cases of pneumonia and save >$3.5 billion in a severe influenza pandemic year.

We also note that the model includes neither the cost nor potential benefits of influenza vaccination at the onset of a pandemic. Assumed pandemic-level rates of influenza in the model are consistent with no vaccination or a vaccine miss-match. If, however, we assume the influenza vaccine is well matched (80% efficacy) with coverage equal to that reported in 2006 [70], severe pandemic incidence would decline from 30% to 24 percent [71]. Under these conditions, the model predicts that vaccination with PCV7 would result in 681,000 cases of pneumonia avoided (compared to 715,000 cases in the base-case) and $6.2 billion in cost savings. Non-pandemic model results are based on recent influenza incidence data and reflect current rates of influenza vaccination.

Finally, it is important to note that *S. pneumoniae *is not the only pathogen known to complicate influenza during a pandemic. In a recent autopsy study of 22 H1N1 victims with bacterial co-infection, 45% were co-infected with *S. pneumoniae*, 32% were co-infected with *S. aureus*, and 27% were co-infected with Group A streptococcus (GAS). It is possible that *S. aureus *or GAS could be the primary cause of bacterial co-infections in a future influenza pandemic, and *S. pneumonaie *could play a less prominent role than in past pandemics. In that case, the benefits of pneumococcal vaccination would be less than those reported in this analysis. Vaccination programs against other pathogens such as *S. aureus *or GAS should be considered as other possibilities for influenza pandemic preparedness.

Our model predicts that routine pneumococcal vaccination is a proactive approach to mitigate effects of a future influenza pandemic, preventing numerous cases of pneumococcal disease and averting >100,000 deaths. The model also highlights the potential to save billions of dollars in health-care costs during an influenza pandemic by avoiding cases of pneumococcal disease and associated health-care utilization. This analysis details additional potential public health and economic benefits of universal vaccination with PCV7, which has already been estimated to be highly cost-effective or cost saving in many countries from various regions of the world [50,62-64]. Countries that have not yet implemented a pneumococcal vaccination program may want to consider it as part of influenza pandemic preparedness.

## Conclusions

The biological plausibility of a link between influenza and increased risk for subsequent pneumococcal disease rests on a number of biological responses to influenza that increase susceptibility to pneumococcal infection [72]. The timing of the exposure to the pneumococcus in relation to the influenza appears to be critical to the outcome in mice-- there is no increased mortality if the pneumococcal exposure precedes influenza, an intermediate risk if exposure is concurrent, and greatly increased risk for a pneumococcal exposure 7 days post-influenza [73]. The likely biological basis for this observation is the effect of γ-interferon, which peaks in mice at 7 days post-influenza and inhibits the ability of alveolar macrophages to clear the pneumococcus [74]. Studies in humans suggest that deaths during the 1918 influenza pandemic were due to complications of bacterial pneumonia [7,8]. Moreover, recent research suggests that pneumococcal conjugate vaccine reduces influenza-associated pneumonia hospitalizations [11].

Given the apparent link between pneumococcal disease and influenza, it is likely that vaccination with PCV7 would reduce the public health burden during an influenza pandemic. Our model predicts that current pneumococcal vaccination practices would substantially reduce the number of cases of all-cause pneumonia and deaths during a severe influenza pandemic by approximately 719,000 and 47,000, respectively. The model also predicts considerable cost savings associated with avoiding pneumococcal disease episodes ($7.3 billion). A large number of the cases avoided are attributable to indirect (herd) effects in the unvaccinated; however, even when indirect (herd) effects are reduced or eliminated, the model still predicts a significant reduction in pneumococcal-related costs during a pandemic.

## Competing interests

DRS is employed by Pfizer; JLR, LJM, KPK, KEG, and MCW served as paid consultants to Pfizer for this study.

## Authors' contributions

DRS and KPK conceived of the study. JLR participated in the study design and methodology, performed the model estimation, and drafted the manuscript. LJM participated in the study design and methodology, participated in estimating model parameters and helped to draft the manuscript. KPK provided clinical input to the study design and vetted model parameter estimation. DRS participated in the design of the study and oversaw study methodology and model estimation. KEG performed the model programming. MCW was a key contributor to model design and vetted the model structure and analyses. All authors read and approved the final manuscript.

## Pre-publication history

The pre-publication history for this paper can be accessed here:

http://www.biomedcentral.com/1471-2334/10/14/prepub
